# Role of actin cytoskeleton in cargo delivery mediated by vertically aligned silicon nanotubes

**DOI:** 10.1186/s12951-022-01618-z

**Published:** 2022-09-08

**Authors:** Yaping Chen, Hao Zhe Yoh, Ali-Reza Shokouhi, Takahide Murayama, Koukou Suu, Yasuhiro Morikawa, Nicolas H. Voelcker, Roey Elnathan

**Affiliations:** 1 Monash Institute of Pharmaceutical Sciences, Monash University, 381 Royal Parade, Parkville, VIC 3052 Australia; 2 Melbourne Centre for Nanofabrication, Victorian Node of the Australian National Fabrication Facility, 151 Wellington Road, Clayton, VIC 3168 Australia; 3 Commonwealth Scientific and Industrial Research Organization (CSIRO), Clayton, VIC 3168 Australia; 4 Institute of Semiconductor and Electronics Technologies, ULVAC Inc, 1220-1 Suyama, Susono, Shizuoka 410-1231 Japan; 5 Department of Materials Science and Engineering, Monash University, 22 Alliance Lane, Clayton, VIC 3168 Australia; 6 INM-Leibnitz Institute for New Materials, Campus D2 2, 66123 Saarbrücken, Germany; 7 School of Medicine, Faculty of Health, Deakin University, Waurn Ponds, Geelong, VIC 3216 Australia; 8 Institute for Frontier Materials, Deakin University, Geelong Waurn Ponds campus, Geelong, VIC 3216 Australia

**Keywords:** Silicon nanotubes, Actin inhibition, Cytoskeleton, Intracellular delivery, Nanoinjection, mRNA, Cytochalasin D, Jasplakinolide

## Abstract

Nanofabrication technologies have been recently applied to the development of engineered nano–bio interfaces for manipulating complex cellular processes. In particular, vertically configurated nanostructures such as nanoneedles (NNs) have been adopted for a variety of biological applications such as mechanotransduction, biosensing, and intracellular delivery. Despite their success in delivering a diverse range of biomolecules into cells, the mechanisms for NN-mediated cargo transport remain to be elucidated. Recent studies have suggested that cytoskeletal elements are involved in generating a tight and functional cell–NN interface that can influence cargo delivery. In this study, by inhibiting actin dynamics using two drugs—cytochalasin D (Cyto D) and jasplakinolide (Jas), we demonstrate that the actin cytoskeleton plays an important role in mRNA delivery mediated by silicon nanotubes (SiNTs). Specifically, actin inhibition 12 h before SiNT-cellular interfacing (pre-interface treatment) significantly dampens mRNA delivery (with efficiencies dropping to 17.2% for Cyto D and 33.1% for Jas) into mouse fibroblast GPE86 cells, compared to that of untreated controls (86.9%). However, actin inhibition initiated 2 h after the establishment of GPE86 cell–SiNT interface (post-interface treatment), has negligible impact on mRNA transfection, maintaining > 80% efficiency for both Cyto D and Jas treatment groups. The results contribute to understanding potential mechanisms involved in NN-mediated intracellular delivery, providing insights into strategic design of cell–nano interfacing under temporal control for improved effectiveness.

**Supplementary Information:**

The online version contains supplementary material available at 10.1186/s12951-022-01618-z.

## Background

Recent advances in nanofabrication have greatly diversified the engineered nano–bio cellular interfaces for biomedical research [1–5]. In particular, vertically aligned nanoneedles (NNs)—such as nanowires (NWs) [6–10], nanostraws (NSs) [11, 12], nanotubes (NTs) [13, 14] and their electroactive analogues [15–18]—have shown to be promising tools for probing and modulating cell behavior [19–24]. Such highly tunable NNs are increasingly used for complex cellular manipulations such as mechanotransduction [6, 25, 26], biosensing [3, 27, 28], immunomodulation [29], in vivo and ex vivo gene editing [30–33], biomolecular extraction and sampling [34, 35], intracellular probing of action potentials [36], and intracellular delivery [31, 37–42].

One key application of NNs is to deliver diverse bioactive cargos into cells and tissues—a process also known as nanoinjection [43–45]. A wide variety of membrane-impermeant and functional biomolecules (e.g., DNA, RNA, and proteins) have been delivered via NN platforms into different types of cells, including hard-to-transfect stem cells, primary neurons, and immune cells [31, 37–41]. Multiple factors can play a role in NN-mediated intracellular delivery, such as NN physico-chemical property [46, 47], cell rigidity, strength of focal adhesion, duration of interfacing, and modality of assisted interfacing [48–51].

Rational design, engineering, and fabrication of NNs’ physical geometry/architecture—either via colloidal self-assembly [52–56] or nanofabrication routes [42, 57, 58]—can offer close spatial control over optimal, localized, interfacial interactions for improving the nanoinjection efficacy into target cells. Enhanced control of nanoinjection is typically achieved by engineering the physical geometry of NN arrays—their tunable topological configuration (porous, solid, hollow) and their shape, density, height, and diameter [43, 45]. For example, the tunable porosity of mesoporous silicon NNs (pore dimensions and density) provides a large surface area, and so a greater cargo loading capacity than solid and non-porous NNs [59]. In parallel, nanoinjection efficacy can depend on the NN chemical composition and mechanical stiffness [42]. Surface functionalization of NNs is also a prime strategy to gain sufficient control over the cargo loading and release, to modulate NN biocompatibility and biodegradability, and to govern the interaction between the device and the cargo as well as with the targeted cells [43].

Many attempts at nanoinjection that are based on NN-mediated penetration can suffer from inconsistency of reported delivery efficiency [17, 43, 60]: while some can achieve excellent delivery efficiency, others yield poor results. This has prompted a move toward applying external stimuli to the plasma membrane. Physically disrupting the membrane can induce transient ‘holes’, increasing cellular permeability through mechanical [61–63], optical [64, 65], acoustic [66, 67], electrical [68–70], and thermoplasmonic perturbation [43–45, 71]. These types of membrane disruptions and perturbations increase variability and flexibility in nanoinjection because they can be applied across a diverse range of cell and cargo types—enabling generation of near-universal cytosolic access and in vitro (and even in vivo) delivery.

Despite the great efforts spent on improving nanoinjection efficacy, the underlying mechanism is still subject to debate. NNs can induce changes in cell morphology, spreading, cytoskeletal arrangement, proliferation, differentiation, protein expression, and endocytic behavior [24, 26, 72–75], influencing the nanoinjection efficacy on interacting cells. Different theories have been proposed for nanoinjection including mechanical penetration [76, 77], membrane permeabilization [18, 71], and NN-enhanced endocytosis [7, 41]. But due to the complexity of mechanical, biochemical, and biophysical cues at the cell–NN interface, it is highly likely that more than one nanoinjection mechanism occurs within a short timeframe. A better understanding of how NNs interact with the targeted biological system is pivotal to create a non-destructive, stable, yet dynamic cell–NN interface that is essential for efficient cargo delivery with minimized cellular perturbation [43].

Importantly, recent studies have reported that cytoskeletal elements—consisting of actin filaments, microtubules, intermediate filaments, and their related proteins—are heavily involved in generating dynamic membrane structures and cell mechanics on NNs, which can influence the mechanisms of gaining cytosolic entry and cargo delivery at different stages of NN interfacing [75, 78]. Typically, the force generated by cell adhesion to a NN, which contracts actomyosin networks, impacts cell plasma membrane tension and permeability [79]. Upon initial interfacing, actin rearrangement supports focal adhesion and local deformation of cells in contact with NNs, promoting lipid permeabilization at the plasma membrane and providing direct access to the internal compartment [80, 81]; while post the establishment of a stabilized actin meshwork at the cell–NN interface, the recruitment of curvature-sensing proteins can cause nanoscale bending and inward budding of the plasma membrane, favoring endocytic process to facilitate cargo internalization [82].

In this study, we investigated the role of actin cytoskeleton in delivering biomacromolecule (mRNA) into mouse fibroblast (GPE86) cells mediated by silicon NTs (SiNTs). Two actin inhibition drugs, cytochalasin D (Cyto D) and jasplakinolide (Jas), were used to induce cytoskeletal dysfunction of GPE86 cells; Cyto D inhibits actin polymerization, whereas Jas inhibits actin depolymerization. The results from confocal microscopy and flow cytometry demonstrated that actin inhibition from 12 h before SiNT-interfacing (pre-interface treatment) significantly reduced the delivery efficiency (17.2% for Cyto D_treated and 33.1% for Jas_treated) of mRNA into GPE86 cells, compared to that from 2 h after cell–SiNT interfacing (post-interface treatment; 85.4% for Cyto D_treated and 81.0% for Jas_treated), while the untreated control remained the highest in mRNA transfection (with 86.9% efficiency). The findings provide insights into the importance of actin cytoskeleton in facilitating SiNT intracellular delivery, particularly within the initial period (≤ 2 h) of cell–SiNT interface establishment.

## Results and discussion

**Fig. 1:**
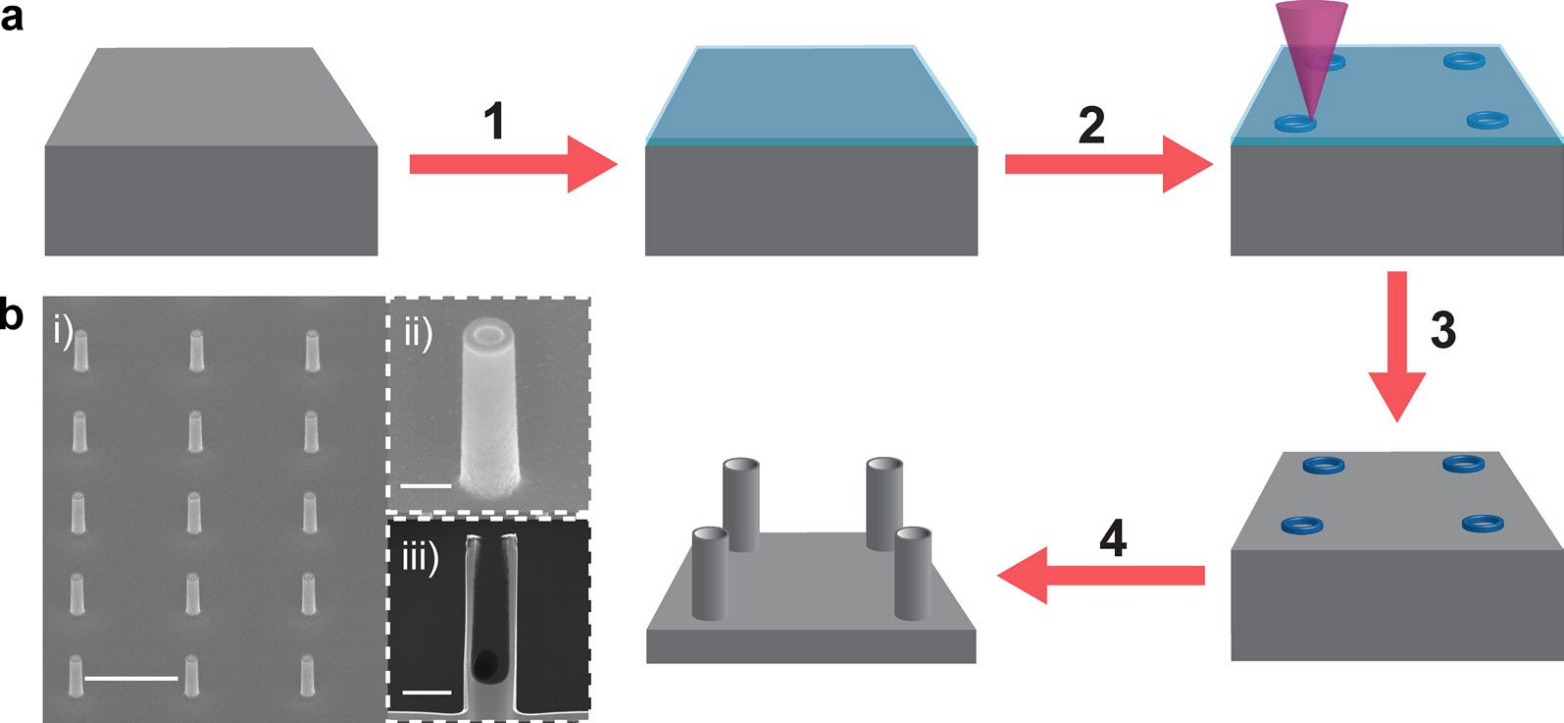
Fabrication of vertically aligned SiNT arrays. **a** Schematics of the SiNT fabrication workflow: (1) Spin coating of HSQ resist on a flat Si wafer; (2) Performance of e-beam lithography (EBL) to write the designed ring patterns within the resist: (3) Chemical development to remove the remaining resist unexposed to EBL; (4) Performance of deep reactive ion etching (DRIE) to obtain SiNT arrays. **b** SEM images showing the (i) zoom-out view, (ii) zoom-in view, and (iii) cross-section after focused ion beam (FIB) miling of SiNTs. Scale bars, (i) 5 µm and (ii, iii) 500 nm

Programmable SiNT arrays were fabricated from a flat Si wafer by resist coating, e-beam lithography (EBL), chemical development, and deep reactive ion etching (DRIE) (Fig. 1a) [13]. The SiNT arrays have precisely controlled geometry (inner/outer diameter of 300/500 nm, height of 2 μm, and pitch of 5 μm; Fig. 1b). The SiNTs are hollow, having an inner cavity of ~ 0.14 μm^3^ (Fig. 1b, iii) that can be used to load controlled amount of biomolecules without any surface functionalization [13]. Once fabricated, the SiNT arrays were cleaned, treated with UV/Ozone to enhance hydrophility, and ethanol sterilized, before loading with fluorescently Cy5-tagged GFP-encoded mRNA (Cy5-mRNA-GFP). The loading of mRNA inside SiNTs was verified by confocal laser microscopy imaging, where Cy5 signals can be observed within the cavity of each SiNT, throughout the entire substrate (Additional file 1: Fig. S1).

To investigate the role of actin cytoskeleton in SiNT-mediated intracellular delivery, we performed a loss-of-function study on adherent mouse embryonic fibroblast (GPE86) cells, as the actin meshwork is crucial for their morphology, adhesion, protrusion formation, and migration [83, 84], which in turn can affect their interfacing with SiNTs and subsequent cargo uptake. GPE86 cells were treated with two actin inhibitors, Cyto D or Jas. Cyto D is a cell permeable fungal toxin that binds to the barbed end of actin filaments, causing the disruption of actin filaments and inhibition of actin polymerization [85]. Jas is a macrocyclic peptide that induces actin polymerization in vitro by stimulating actin filament nucleation; it binds to the side of actin filaments and inhibits polymer disassembly (depolymerization); it also competes with phalloidin for actin binding [86].

To find the optimal treatment conditions of Cyto D and Jas (i.e., sufficient to induce actin inhibition while maintaining high cell viability), we titrated the drug concentrations from 0.0625 to 4.0 μM for GPE86 cell treatment. By staining the cytoskeletal elements F-actin and vinculin—using phalloidin and anti-vinculin antibody, respectively — together with Hoechst (nucleus stain), we were able to observe the morphology changes of GPE86 cells under different treatment conditions using confocal microscopy. The imaging demonstrated that treatment of Cyto D at concentrations ≥ 2.0 μM significantly altered the actin structure and cytoskeleton meshwork of GPE86 cells, whereas concentrations ≥ 0.25 μM was required for Jas to override phalloidin for F-actin binding (Additional file 1: Fig. S2a–c). Live/dead staining using fluorescein diacetate (FDA, cell-permeable dye staining live cell) and propidium iodide (PI, cell-impermeable dye staining dead cells) confirmed that GPE86 cells treated with 2.0 μM Cyto D and 0.25 μM Jas maintained high viability similar to that of untreated control (> 90%) after 24 h (Additional file 1: Fig. S2d). Therefore, we decided to apply these two optimal drug treatments (2.0 μM Cyto D and 0.25 μM Jas) for inhibiting actin and to study its role in SiNT-mediated mRNA delivery.

**Fig. 2:**
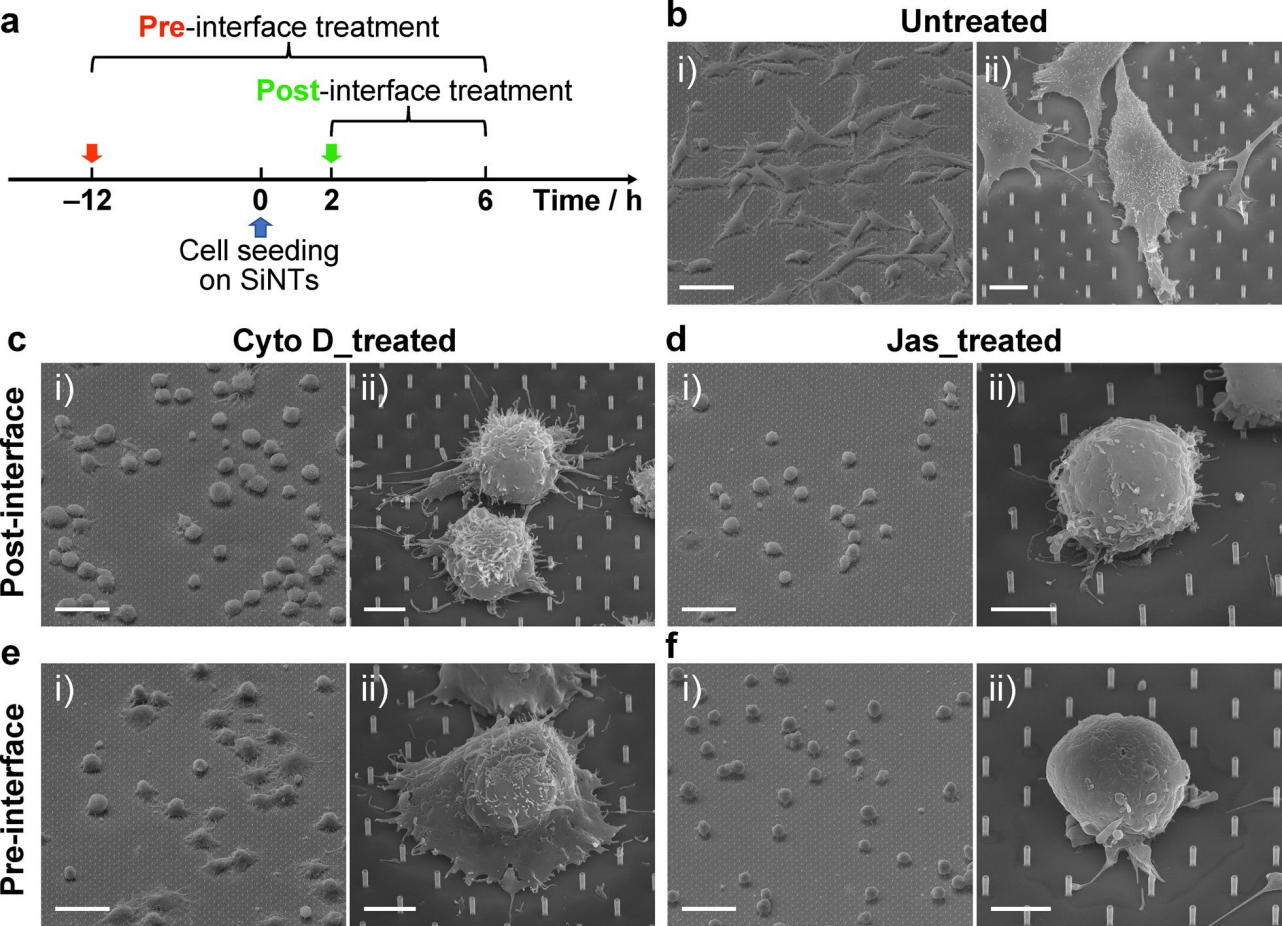
Effects of actin inhibition on cell morphology on SiNTs. **a** Schematic of applying actin inhibitors (Cyto D or Jas) 12 h before or 2 h after SiNT interfacing, for pre- or post-interface treatment, respectively. **b–f** SEM images showing (i) zoom-out and (ii) zoom-in views of (b) untreated, and (c,e) Cyto D_treated and (d,f) Jas_treated GPE86 cells under pre- or post-interface treatment. Scale bars, (i) 40 µm and (ii) 5 µm

To determine the time window when actin predominately responds to SiNT-driven stimuli and whether abnormal changes in actin organization affect subsequent mRNA delivery, we compared the effects of actin inhibition before and after the establishment of cell–SiNT interfacing (Fig. 2a). For “pre-interface” treatment, GPE86 cells were treated with Cyto D or Jas 12 h before seeding onto SiNTs, and the treatment continued throughout the entire 6 h interfacing period; for “post-interface” treatment, Cyto D or Jas was added into the culture media 2 h after GPE86 cell seeding on SiNTs, and the treatment lasted for 4 h till the end of cell–SiNT interfacing. The samples containing cells were then processed for confocal microscopy and scanning electron microscopy (SEM); alternatively, cells were detached from the SiNT substrates by trypsinization and analyzed by means of flow cytometry.

SEM images illustrated the distinct morphology changes of GPE86 cells under different treatment conditions (Fig. 2d–f). Untreated cells were extensively spreading on the SiNT array, generating long filopodia and short lamellipodia protrusions that enhance the contact and focal adhesion on SiNTs (Fig. 2b) [87]. However, Fig. 2c–f show that the cell morphology was altered remarkably after addition of the two types of actin inhibitors. The membrane of GPE86 cells receiving Cyto D treatment failed to spread on SiNTs, despite the observation of a limited number of irregular filopodia and lamellipodia for post- and pre-interface treatment, respectively (Fig. 2c, e); the remaining of these abnormal protrusions can be attributed to Cyto D treatment, which inhibits both the association and dissociation of actin subunits [88]. Jas treatment, on the other hand, significantly impaired cell attachment and spreading on SiNTs; the cells exhibited spherical morphology, sitting on top of the array with merely a few fibrous membrane branches attached to the SiNTs for both post- and pre-interface treatment (Fig. 2d, f). In addition, by using FIB-SEM, we were able to observe a cross-sectional interface between the cell and the individual SiNTs. In the control group, it was clear that SiNTs remained intact after cell interfacing, but in pre- and post-interface treatment groups, some SiNTs were found bended or broken (Additional file 1: Fig. S3); this is likely due to the actin inhibition that can impact the contractility of the cytoskeleton network and membrane motility, causing the buckling of SiNTs that have been in contact with the cell [89, 90].

**Fig. 3:**
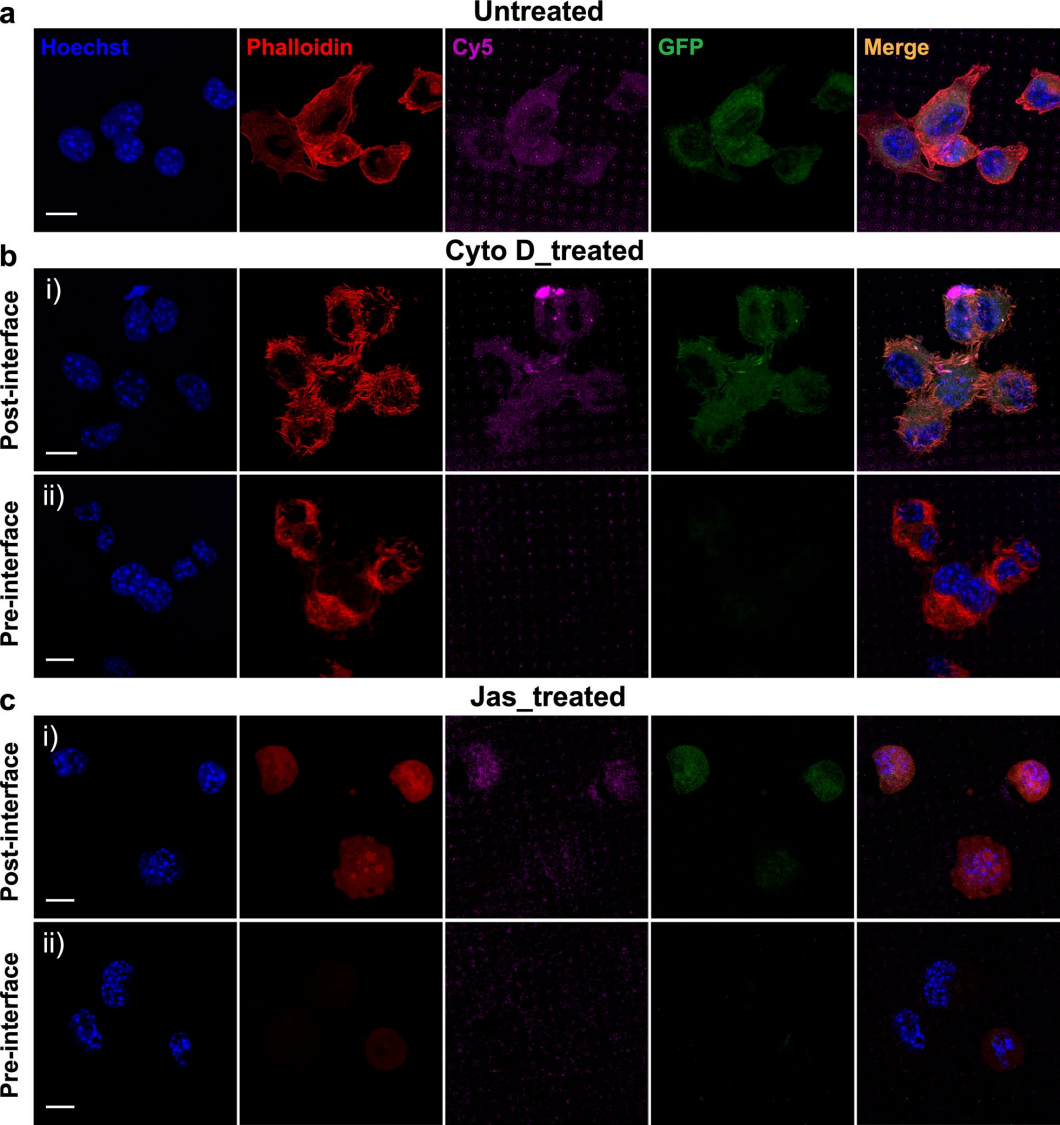
Effects of actin inhibition on SiNT-mediated mRNA delivery. Confocal images of **a** untreated GPE86 cells, and cells with pre- or post-interface treatment of **b** Cyto D or **c** Jas on Cy5 (magenta)-mRNA-GFP (green) loaded SiNTs after 6 h interfacing. Cells were stained with Hoechst (blue) and phalloidin (red) to indicate the nucleus and F-actin, respectively. Scale bars, 10 µm

We next investigated whether the actin dysfunction can influence SiNT-mediated mRNA delivery. Using confocal microscopy and flow cytometry, we were able to detect Cy5 and GFP signals within the cells on and off SiNT arrays; Cy5 indicates mRNA insertion into the cells, and GFP indicates the preserved bioactive function of mRNA that leads to protein translation/expression. Figure 3a and Additional file 1: S4a shows the observation of both Cy5 and GFP signals in untreated GPE86 cells, which maintained undisrupted F-actin meshwork indicated by phalloidin staining. Interestingly, both signals were found, similar to that in control, in cells receiving post-interface treatment with Cyto D and Jas, despite the significant changes in their F-actin structures (Fig. 3b, c, Additional file 1: Fig. S4b, c). But it is evident that Cy5 and GFP signals were barely detectable in cells with pre-interface actin inhibition (Fig. 3b,c, Additional file 1: Fig. S4b, c). The absence of phalloidin staining in Fig. 3c, ii was due to the strong competition of actin binding by pre-interface Jas treatment.

**Fig. 4:**
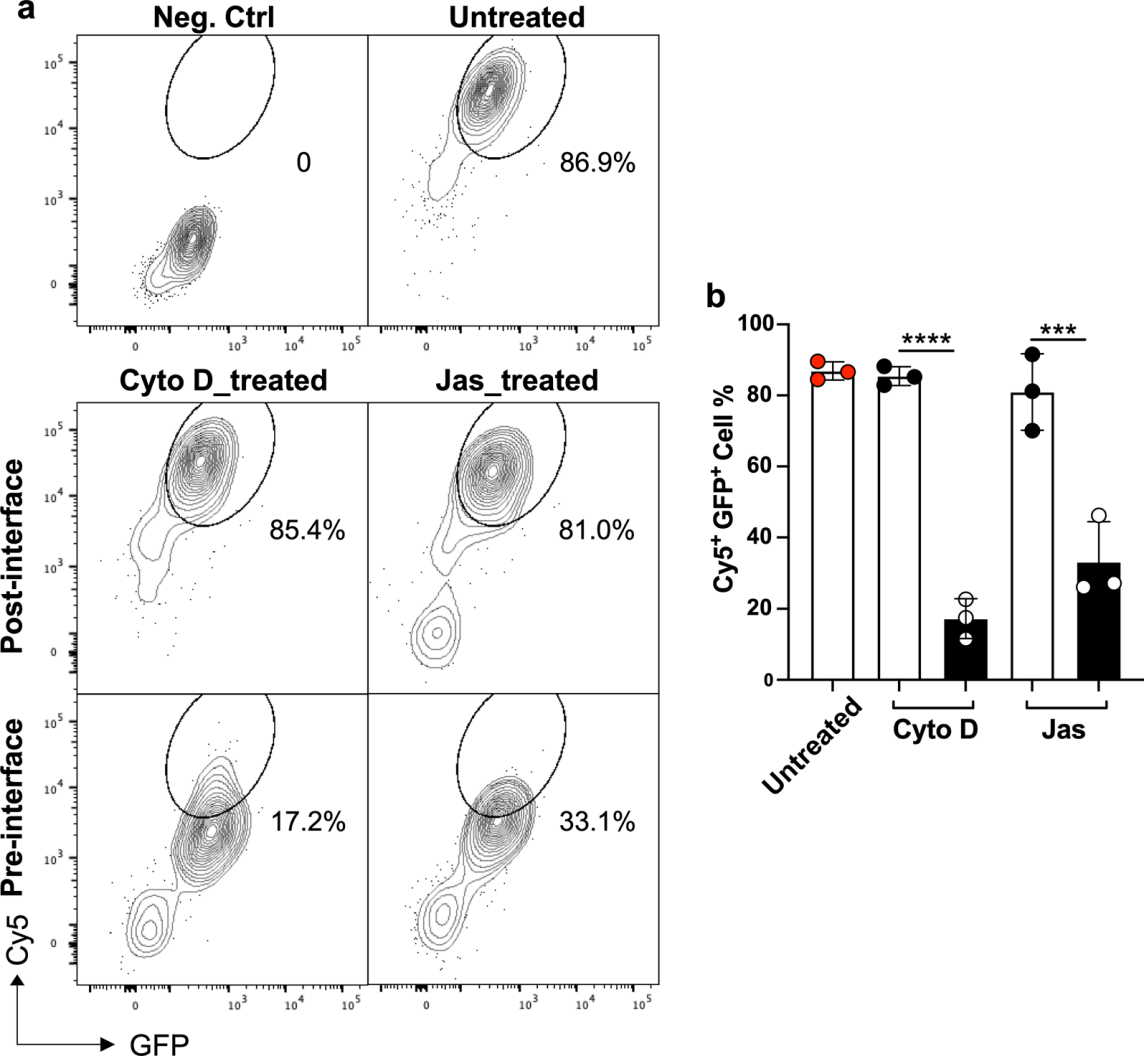
Quantitative analysis of SiNT-mediated mRNA delivery efficiency under different treatment conditions. **a** Flow cytometry analysis showing the gating of Cy5^+^ GFP^+^ population within untreated and pre-/post-interface Cyto D/Jas treated GPE86 cells after detachment from SiNTs. **b** Quantification of Cy5^+^ GFP.^+^ populations within detached GPE86 cells as in (**b**). ***p ≤ 0.0001, ****p < 0.0001 (One-way ANOVA). n = 3

Flow cytometry analysis of detached cells provided quantitative results of mRNA transfection efficiency under each condition, which were in line with the findings from confocal microscopy. There was no significant difference among untreated and post-interface Cyto D/Jas-treated cells, with transfection efficiencies of 86.9%, 85.4%, and 81.0%, respectively (Fig. 4). However, pre-interface Cyto D treatment led to a dramatic reduction in transfection efficiency to 17.2%, with pre-interface Jas treatment slightly higher at 33.1%.

## Conclusion

In this study, we demonstrated that actin dysfunction, by either inhibiting polymerization or depolymerization, prior to SiNT-interfacing profoundly impacts SiNT-mediated intracellular delivery of mRNA. Nevertheless, once a stable cell–SiNT interface has been established, the inhibition on actin dynamics and cytoskeleton does not significantly reduce the mRNA transfection efficiency; this also indicates that SiNT-mediated cargo delivery mainly occurs at the initial (≤ 2 h) interfacing period. The findings not only help understand the role of actin during SiNT-interfacing and intracellular delivery, but also provide information for strategic decision making when engineering cell–nanostructure interfaces for other biological applications especially that are time-sensitive and involve cytoskeletal rearrangement.

## Experimental section

### SiNT fabrication

(1) Substrate cleaning: Flat silicon wafers (4″, p-type, 3–6 Ω cm, < 100 > , Siltronix, France) were cleaned by sonication in 1:1 solution of ethanol:acetone for 5 min and then sonicated again in MilliQ water for 5 min. This was followed by dipping the wafers into boiling Piranha solution (3:1 H_2_SO_4_:H_2_O_2_ v/v, 75 °C, Avantor Performance Materials) for 1 h to remove any organic contaminants, then washing with water and drying under a nitrogen jet. (2) E-beam lithography (EBL): HSQ resist (XR-1541–002, Dow Corning, USA) was spin coated onto a silicon wafer with a spin speed of 1500 rpm and an acceleration of 300 rpm/s for 1 min. The sample was directly loaded into an EBL system (VISTEC EBPG-5000 + , Raith Company, Germany) without baking. The EBL was performed at an accelerating voltage of 100 kV with a beam current of 30 nA, using a dose of 1000 μCcm^−2^. After electron-beam exposure, the HSQ resist was developed using AZ 726 MIF. Development was stopped with water and samples were dried under a nitrogen jet. (3) Deep reactive ion etching (DRIE): Samples prepared by EBL were inserted into an ULVAC NLD5700 DRIE. Silicon etching was performed in a simultaneous flow of SF_6_ and O_2_ at a pressure of 1 Pa with Antenna RF power of 200 W and Bias RF LF power of 16 W. He pressure was set at 2000 Pa and the circulator at 20 °C. The etching time was 145 s.

### Loading of mRNA onto SiNTs

10 µL of Cy5-GFP-mRNA (200 ng µL^–1^, Trilink Biotechnologies) was placed on the SiNT substrates and allowed to stand 1 h. Excess mRNA was removed from the substrates before seeding cells. Each NT was filled with ~ 2.8 × 10^−8^ ng mRNA, with each substrate containing ~ 0.028 ng mRNA.

### Cell culture

GPE86 cells (ATCC, mouse embryonic fibroblasts) were grown and maintained in complete Dulbecco’s modified Eagle’s medium (DMEM (Gibco), supplemented with 10% fetal bovine serum (FBS, Gibco), 1 mM sodium pyruvate, 2 mM L-glutamine, 100 U mL^−1^ penicillin, and 100 μg mL^−1^ streptomycin (Gibco). The cells were incubated at 37 °C with 5% CO_2_.

### Actin inhibitor treatment

GPE86 cells were treated with actin inhibitors (Cyto D, 2.0 µM; Jas, 0.25 µM) 12 h before or 2 h after SiNT interfacing, termed pre- and post-interface treatment, respectively. Untreated cells served as control.

### SiNT-mediated mRNA delivery

Untreated and Cyto D-/Jas-treated (pre-interface) GPE86 cells were seeded onto SiNT substrates (5 mm × 5 mm) loaded with Cy5-mRNA-GFP in 48-well plate (25,000 cells/well, in 250 μL Opti-MEM (Gibco)), followed by centrifugation at 250 *g*, 32 °C, for 15 min. After centrifugation and 2 h incubation at 37 °C, 5% CO_2_, substrates carrying GPE86 cells were transferred to new plates; untreated cells were cultured in fresh complete DMEM; cells with pre- and post-interface treatment were cultured in fresh complete DMEM containing Cyto D or Jas for a further 4 h. (1) For fluorescence microscopy imaging, cells grown on substrates were rinsed with DPBS and fixed in a solution of 4% paraformaldehyde (PFA; Electron Microscopy Sciences) for 10 min, followed by permeabilization in 0.1% Triton X-100 (Sigma-Aldrich) in DPBS for 5 min at RT. After washing three times with DPBS, cells were stained with relevant fluorescence markers before proceeding to microscopy imaging. (2) For flow cytometry analysis, cells on substrates were trypsinized with 0.25% Trypsin–EDTA (Gibco), neutralized with DMEM, transferred to v-bottom 96-well plate, spun down, and washed twice with flow cytometry staining buffer (FACS buffer). Cells were stained with relevant fluorescence markers before proceeding to flow cytometry detection.

### Laser scanning confocal microscopy

A Leica Stellaris 5 confocal laser scanning microscope system was used for fluorescence imaging. Hoechst, GFP, Alexa Fluor 568 Phalloidin, Cy5 were excited at 340, 493, 578, and 650 nm, with emission at 460, 515, 636, and 670 nm respectively. A 20 × dry objective lens and 60 × oil immersed objective lens were used for observation and more than 3 different locations were selected for 3 samples. Images were analyzed using Leica Application Suite X provided by the manufacturers and ImageJ.

### Flow cytometry

An LSR Fortessa X20 flow cytometer (BD) was used to investigate the insertion and transfection efficiency of cells harvested from the substrates.

### Flow cytometry insertion and transfection efficiency

To detect the insertion of mRNA and GFP expression, GPE86 were harvested from the substrates loaded with mRNA after 6 h incubation. The excitation/emission wavelengths for Cy5 and GFP on LSR Fortessa X20 were 678/694, and 488/540 nm respectively. Proper negative and positive controls were used for the flow cytometry analysis. Compensation was done to avoid fluorescence leakage between different channels.

### Sample preparation for SEM imaging

Cells grown on SiNT substrates were rinsed with 0.1 M sodium cacodylate buffer (Electron Microscopy Sciences) and fixed with 2.5% glutaraldehyde (Electron Microscopy Sciences) in 0.1 M sodium cacodylate at 4 °C overnight. Following this, substrates were washed (3 × 5 min) with chilled 0.1 M sodium cacodylate buffer and post-fixed with 1% osmium tetroxide (Electron Microscopy Sciences) in 0.1 M sodium cacodylate at RT for 1 h. After repeating the washing step, substrates were gradually dehydrated with increasing concentrations of ethanol; 50%, 70%, 90% (1 × 10 min) and 100% (2 × 10 min) at RT, and finally were critical point dried (CPD 030 Critical Point Dryer, BAL-TEC). Substrates were then mounted on SEM stubs and sputter coated with a 7 nm layer of either gold or platinum in order to increase their conductivity.

### SEM imaging

SEM imaging of both bare SiNTs and SiNT substrates with cells was performed on a Nova NanoSEM 430 (FEI). The images were taken at tilt (45˚) or top views with an electron beam acceleration voltage of 3–5 kV and a current of 80 pA, while using a secondary electron detector.

### Intracellular compartments staining and FIB-SEM sample preparation

Heavy metal staining and resin embedding were used as the sample preparation method. Samples were rinsed with 0.1 M sodium cacodylate buffer (Electron Microscopy Sciences) and fixed with 2.5% glutaraldehyde (Electron Microscopy Sciences) in the same buffer at 4 °C for 1 h. Following this, the samples were washed (3 × 5 min) with chilled 0.1 M sodium cacodylate buffer and quenched with chilled 20 × 10^−3^ M glycine solution (Sigma-Aldrich) in the same buffer for 20 min. After repeating the washing step, samples were post fixed by combining equal volumes of 4% aqueous osmium tetroxide with 2% potassium ferrocyanide (UNIVAR) in 0.2 M sodium cacodylate buffer on ice for 1 h. Samples were re-washed (3 × 5 min) with chilled buffer and incubated with 1% tannic acid (BDH) in deionized water at RT for 20 min. After rinsing with sodium cacodylate buffer (2 × 5 min), samples were further incubated with 2% aqueous osmium tetroxide at RT for 30 min. Samples were washed (2 × 5 min) with deionized water and incubated with syringe-filtered 4% aqueous uranyl acetate (UNIVAR) at 4 °C overnight. Samples were washed (3 × 5 min) with chilled deionized water and gradually dehydrated with increasing concentrations of ethanol: 10%, 30%, 50%, 70%, 90%, and 100% (1 × 7 min) at RT. An Epon 812 resin 20 mL solution was prepared by initially mixing 12.2 g of DDSA (dodecenyl succinic anhydride specially distilled, Electron Microscopy Sciences), 4.4 g of Araldite (GY 502, Electron Microscopy Sciences), and 6.2 g of Procure 812 (EMBED 812 RESIN) using a mechanical stirrer. Once the solution was uniformly mixed, 0.8 mL of BDMA (N-benzyldimethylamine, Electron Microscopy Sciences) was added while stirring. Samples were infiltrated with increasing concentrations of the freshly prepared resin solution in 100% ethanol at RT and in a sealed container using the following ratios: 1:3 (3 h), 1:2 (3 h), 1:1 (overnight), 2:1 (3 h), 3:1 (3 h). Following this, samples were finally infiltrated with 100% resin solution overnight. The excess resin was drained away by mounting the samples vertically for 1 h and samples were left for polymerization at 60 °C in an oven for 48 h. The sample were sputtered coated with 10 nm Au prior sectioning and imaging.

### FIB sectioning and imaging

FIB sectioning was performed using a Thermo Fischer Helios Nanolab 600. Prior to FIB sectioning, ion-beam facilitated Pt deposition of ~ 0.5 µm thickness was performed to protect the area of interest at 30 kV and with 3–5 pA µm^–2^ current density. Rough milling was performed at an acceleration voltage of 30 kV voltage and current ranging between 2.8 and 4.6 nA, and the surface was polished with 30 kV voltage and 0.46–2.8 nA. Images were taken using an electron beam acceleration voltage of 5 and 10 kV and current of 86 nA using free field and immersion mode with through-the-lens detector (TLD) operating under secondary electron collection mode, at dwell time of 5 µs and 6144 × 4096 pixel^2^ resolution. Original images are black–white inverted.

### Data processing and statistical analysis

Fluorescence and SEM images were processed and analyzed by Image J. Flow cytometry data were analyzed with FlowJo. All statistical analysis was performed using Prism GraphPad 9. Non-parametric two-sided Mann–Whitney U-tests were performed for comparison between two groups. A one-way ANOVA was used to calculate univariate data set with more than two groups.

## Supplementary Information

**Additional file 1:** **Figure S1.** Loading Cy5-mRNA-GFP onto SiNT arrays. Representative confocal microscopy images showing top views, (a) zoom-out and (b) zoom-in, and (c) 3D view of SiNTs loaded with Cy5-mRNA-GFP (magenta). **Figure S2.** Titration of optimal condition for actin inhibitor treatment. (**a–c**) Confocal images of (**a**) untreated cells, and cells treated with (**b**) Cyto D and (c) Jas at different concentrations from 0.0625 to 4.0 µM. Cells were stained with Hoechst (blue), phalloidin (red), and vinculin (green) for the nucleus, F-actin, and cytoskeletal elements, respectively. Red squares indicate the threshold concentration required to induce sufficient actin inhibition for Cyto D and Jas. Scale bars, 10 µm. (**d**) Fluorescence images showing live/dead staining by Hoechst (blue), FDA (green, live cells), and PI (red, dead cells) of untreated cells and cells treated with Cyto D (2.0 µM) or Jas (0.25 µM). (**e**): Quantification of cell viability of the untreated and Cyto D/Jas-treated cells as in d. Scale bars, 100 µm. n =3. **Figure S3.** Effects of actin inhibition on cell–SiNT interface. FIB-SEM images of (**a**) untreated, and (**b,d**) Cyto D_treated and (**c,e**) Jas_treated GPE86 cells under pre- or post-interface treatment; (ii) are enlarged views of insets from (i). Red arrows indicate broken SiNTs. Scale bars, (i) 5 µm and (ii) 1 µm. **Figure S4.** Effects of actin inhibition on SiNT-mediated mRNA delivery. Confocal images of (**a**) untreated GPE86 cells, and cells with pre- or post-interface treatment of (**b**) Cyto D or (**c**) Jas on Cy5 (magenta)-mRNA-GFP (green) loaded SiNTs after 6 h interfacing. Cells were stained with Hoechst (blue) and phalloidin (red) to indicate the nucleus and F-actin, respectively. Scale bars, 20 µm.

## Data Availability

All data generated or analyzed during this study are included in this published article (and its additional files).
