# Supplementary material for: Role of actin cytoskeleton in cargo delivery mediated by vertically aligned silicon nanotubes
Source: J Nanobiotechnology. 2022 Sep 8;20:406. doi: 10.1186/s12951-022-01618-z (PMC9461134; doi:10.1186/s12951-022-01618-z)
Supplement: Supplementary file 1 — Additional file 1: Figure S1. Loading Cy5-mRNA-GFP onto SiNT arrays. Representative confocal microscopy images showing top views, (a) zoom-out and (b) zoom-in, and (c) 3D view of SiNTs loaded with Cy5-mRNA-GFP (magenta). Figure S2. Titration of optimal condition for actin inhibitor treatment. (a–c) Confocal images of (a) untreated cells, and cells treated with (b) Cyto D and (c) Jas at different concentrations from 0.0625 to 4.0 µM. Cells were stained with Hoechst (blue), phalloidin (red), and vinculin (green) for the nucleus, F-actin, and cytoskeletal elements, respectively. Red squares indicate the threshold concentration required to induce sufficient actin inhibition for Cyto D and Jas. Scale bars, 10 µm. (d) Fluorescence images showing live/dead staining by Hoechst (blue), FDA (green, live cells), and PI (red, dead cells) of untreated cells and cells treated with Cyto D (2.0 µM) or Jas (0.25 µM). (e): Quantification of cell viability of the untreated and Cyto D/Jas-treated cells as in d. Scale bars, 100 µm. n =3. Figure S3. Effects of actin inhibition on cell–SiNT interface. FIB-SEM images of (a) untreated, and (b,d) Cyto D_treated and (c,e) Jas_treated GPE86 cells under pre- or post-interface treatment; (ii) are enlarged views of insets from (i). Red arrows indicate broken SiNTs. Scale bars, (i) 5 µm and (ii) 1 µm. Figure S4. Effects of actin inhibition on SiNT-mediated mRNA delivery. Confocal images of (a) untreated GPE86 cells, and cells with pre- or post-interface treatment of (b) Cyto D or (c) Jas on Cy5 (magenta)-mRNA-GFP (green) loaded SiNTs after 6 h interfacing. Cells were stained with Hoechst (blue) and phalloidin (red) to indicate the nucleus and F-actin, respectively. Scale bars, 20 µm. [file 12951_2022_1618_MOESM1_ESM.docx]

**Supplementary information**

**Role of actin cytoskeleton in cargo delivery mediated by vertically aligned silicon nanotubes**

Yaping Chen^1,2^*, Hao Zhe Yoh^1,2,3^, Ali-Reza Shokouhi^1,2^, Takahide Murayama^4^, Koukou Suu^4^, Yasuhiro Morikawa^4^, Nicolas H. Voelcker^1,2,3,5,6^*, Roey Elnathan^1,2,7,8^*

^1^Monash Institute of Pharmaceutical Sciences, Monash University, 381 Royal Parade, Parkville, VIC 3052, Australia

^2^Melbourne Centre for Nanofabrication, Victorian Node of the Australian National Fabrication Facility, 151 Wellington Road, Clayton, VIC 3168, Australia

^3^Commonwealth Scientific and Industrial Research Organization (CSIRO), Clayton, VIC 3168, Australia

^4^Institute of Semiconductor and Electronics Technologies, ULVAC Inc., 1220-1 Suyama, Susono, Shizuoka, 410-1231, Japan

^5^Department of Materials Science and Engineering, Monash University, 22 Alliance Lane, Clayton, VIC 3168, Australia.

^6^INM-Leibnitz Institute for New Materials, Campus D2 2, Saarbrücken 66123, Germany

^7^School of Medicine, Faculty of Health, Deakin University, Waurn Ponds, 3216, Australia

^8^Institute for Frontier Materials, Deakin University, Geelong Waurn Ponds campus, VIC, 3216, Australia

**
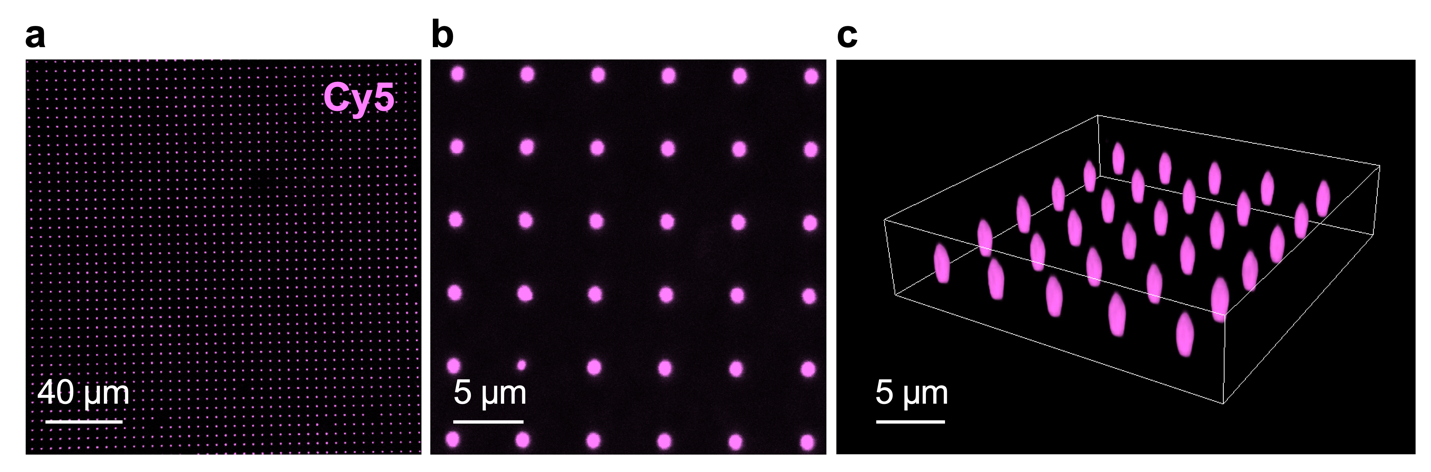
Figure S1: Loading Cy5-mRNA-GFP onto SiNT arrays.** Representative confocal microscopy images showing top views, (a) zoom-out and (b) zoom-in, and (c) 3D view of SiNTs loaded with Cy5-mRNA-GFP (magenta).

**
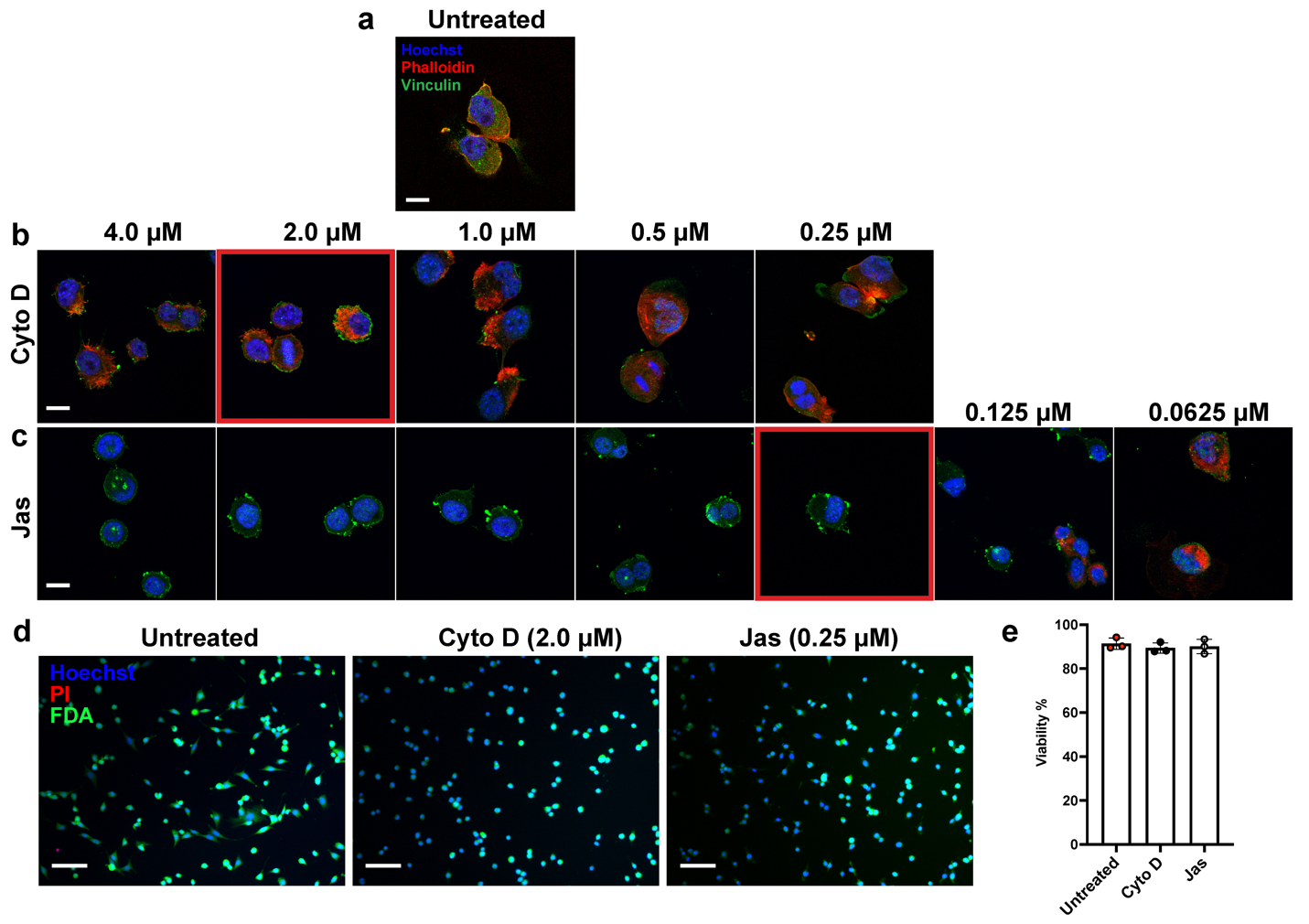
Figure S2: Titration of optimal condition for actin inhibitor treatment.** (**a–c**) Confocal images of (**a**) untreated cells, and cells treated with (**b**) Cyto D and (c) Jas at different concentrations from 0.0625 to 4.0 µM. Cells were stained with Hoechst (blue), phalloidin (red), and vinculin (green) for the nucleus, F-actin, and cytoskeletal elements, respectively. Red squares indicate the threshold concentration required to induce sufficient actin inhibition for Cyto D and Jas. Scale bars, 10 µm. (**d**) Fluorescence images showing live/dead staining by Hoechst (blue), FDA (green, live cells), and PI (red, dead cells) of untreated cells and cells treated with Cyto D (2.0 µM) or Jas (0.25 µM). (**e**): Quantification of cell viability of the untreated and Cyto D/Jas-treated cells as in d. Scale bars, 100 µm. n =3

**
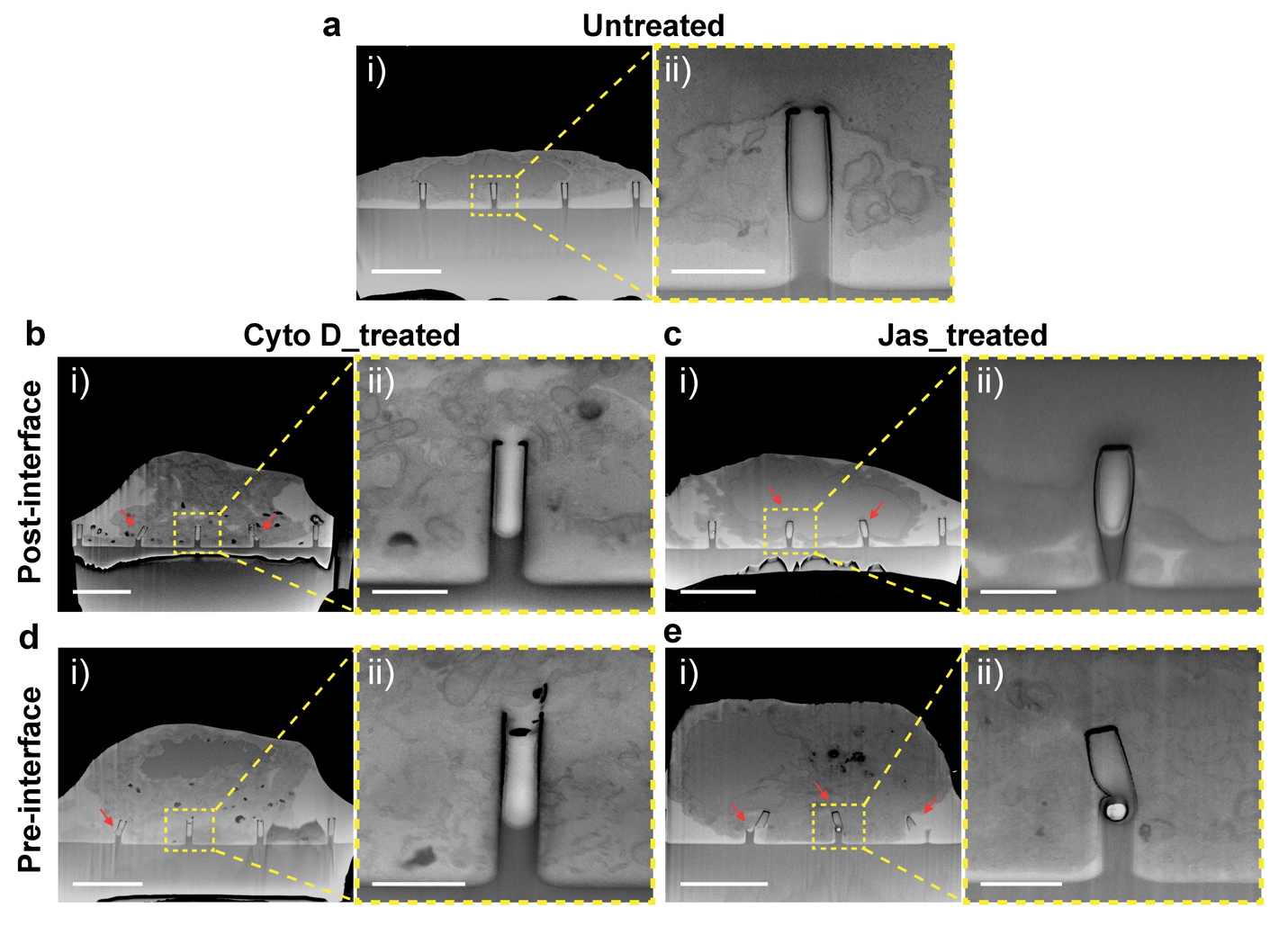
**

**Figure S3:** **Effects of actin inhibition on cell–SiNT interface.** FIB-SEM images of (**a**) untreated, and (**b,d**) Cyto D_treated and (**c,e**) Jas_treated GPE86 cells under pre- or post-interface treatment; (ii) are enlarged views of insets from (i). Red arrows indicate broken SiNTs. Scale bars, (i) 5 µm and (ii) 1 µm.

**
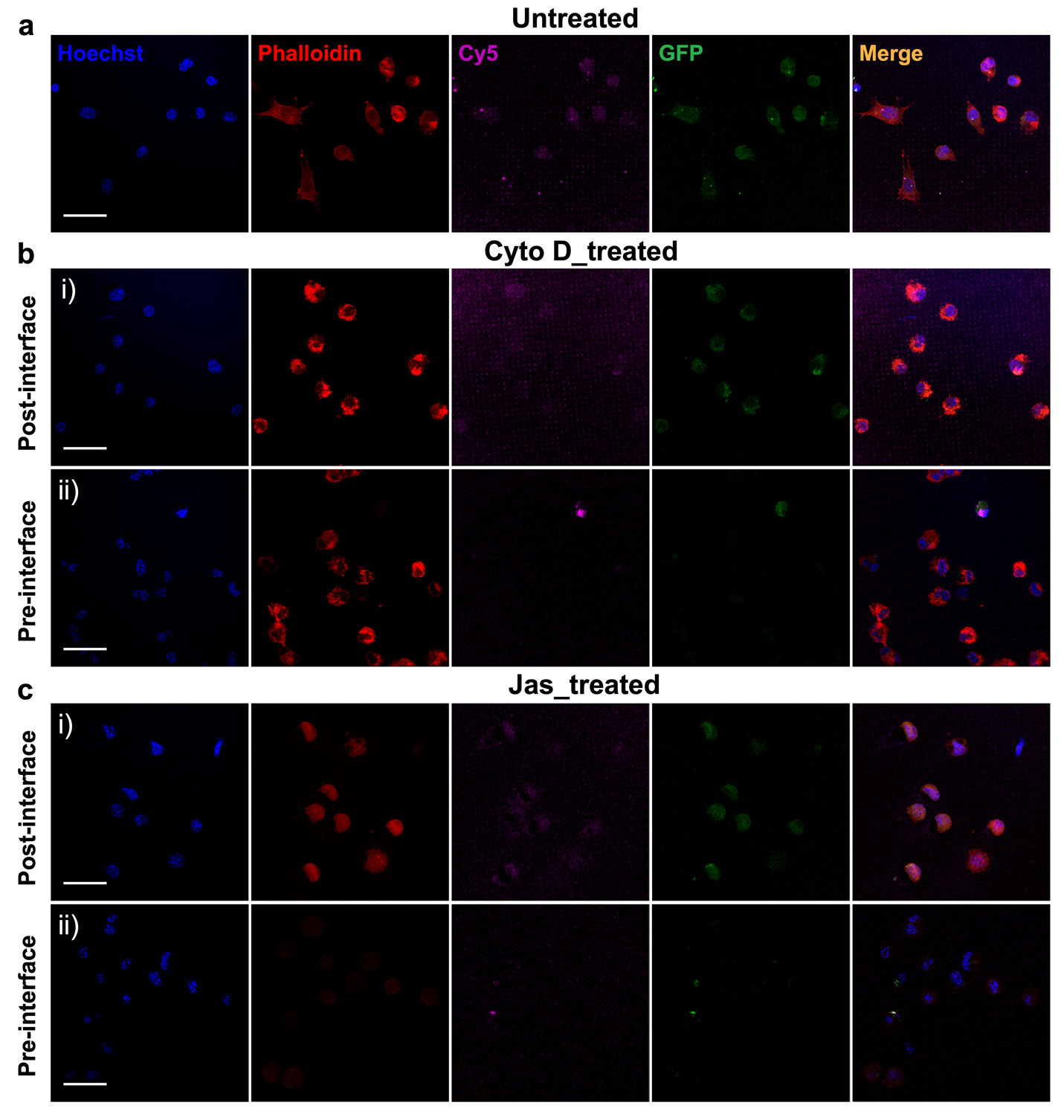
Figure S4:** **Effects of actin inhibition on SiNT-mediated mRNA delivery.** Confocal images of (**a**) untreated GPE86 cells, and cells with pre- or post-interface treatment of (**b**) Cyto D or (**c**) Jas on Cy5 (magenta)-mRNA-GFP (green) loaded SiNTs after 6 h interfacing. Cells were stained with Hoechst (blue) and phalloidin (red) to indicate the nucleus and F-actin, respectively. Scale bars, 20 µm.
